# Revealing the pulse-induced electroplasticity by decoupling electron wind force

**DOI:** 10.1038/s41467-022-34333-2

**Published:** 2022-10-31

**Authors:** Xing Li, Qi Zhu, Youran Hong, He Zheng, Jian Wang, Jiangwei Wang, Ze Zhang

**Affiliations:** 1 Center of Electron Microscopy and State Key Laboratory of Silicon Materials, School of Materials Science and Engineering, Zhejiang University, Hangzhou, 310027 China; 2 School of Physics and Technology, Center for Electron Microscopy, MOE Key Laboratory of Artificial Micro- and Nano-structures, and Institute for Advanced Studies, Wuhan University, Wuhan, 430072 China; 3 Department of Mechanical and Materials Engineering, University of Nebraska-Lincoln, Lincoln, NE 68583 USA; 4 Wenzhou Key Laboratory of Novel Optoelectronic and Nano Materials, Institute of Wenzhou, Zhejiang University, Wenzhou, 325006 China

**Keywords:** Electronic devices, Electronic properties and materials, Metals and alloys

## Abstract

Micro/nano electromechanical systems and nanodevices often suffer from degradation under electrical pulse. However, the origin of pulse-induced degradation remains an open question. Herein, we investigate the defect dynamics in Au nanocrystals under pulse conditions. By decoupling the electron wind force via a properly-designed in situ TEM electropulsing experiment, we reveal a non-directional migration of Σ3{112} incoherent twin boundary upon electropulsing, in contrast to the expected directional migration under electron wind force. Quantitative analyses demonstrate that such exceptional incoherent twin boundary migration is governed by the electron-dislocation interaction that enhances the atom vibration at dislocation cores, rather than driven by the electron wind force in classic model. Our observations provide valuable insights into the origin of electroplasticity in metallic materials at the atomic level, which are of scientific and technological significances to understanding the electromigration and resultant electrical damage/failure in micro/nano-electronic devices.

## Abstract (web summary)

The origin of the degradation of nanodevices under electrical pulses still remains unknown. Here, Li et al. reveal the mechanism of direct electron-dislocation interaction by decoupling the electron wind force using in situ electropulsing, providing atomistic insight into the electroplasticity.

## Introduction

Integration and performance of Micro/Nano Electromechanical Systems (MEMS/NEMS) can be greatly improved by the miniaturization of electronic devices. Associated with the device miniaturization, however, is the increased current density in its nanosized components, which can gradually degrade the circuits by heat generation^1,2^, void formation^3,4^, creep^5^ and eventually result in the failure of electronic devices. Numerous experiments have been conducted to investigate the degradation of electronic devices under steady current conditions^1,6^, with special focus on the electromigration induced-damage. In real applications of nanodevices (such as phase change memory), however, current signals are usually applied in a pulsed modality^7^. The overloaded pulse current, as well as continuous-applied current shock, could result in significant structural changes of the material^8–10^, leading to catastrophic failure of nanodevices^11^. Despite these notorious degradation phenomena, the underlying mechanism of pulsed current effects have not been fully understood.

On the other hand, high-energy electrical pulse processing is an efficient approach to modify the microstructures of materials and tailor their mechanical properties^8,9,12–14^. A number of studies showed that the formability of metallic materials, including steels^15,16^, titanium alloys^14,17^, and magnesium alloys^18^ can be improved significantly by high-energy electrical pulse processing, which were often ascribed to the electroplasticity. Based on experimental measurements, theoretical models of electroplasticity have been proposed and developed since 1970s^19^. It was believed that the drifting electrons can transfer their momentums to atoms, leading to enhanced mobility of defects or interfaces, i.e., the so-called electron wind force theory^20,21^. This theory can explain some experimental observations about electroplasticity^7,21^; however, a direct relation between the dislocation motion and electron wind force remains unestablished due to the interference of high density of defects and the absence of experimental observation interlinking the dislocation motion and electron wind force (e.g., directional dislocation motion along the direction of electron wind force^7,21^). Besides, a recent study by Zhao et al.^22^ reported that the application of pulsed current can substitutionally change the slip patterns of dislocations in Ti-7Al alloy, while the electron wind force shows a negligible contribution to the dislocation motion. These controversies suggest the possible impact of other athermal effects of pulsed current on electroplasticity, e.g., direct electron activation of dislocations proposed in some studies^12,23^, beyond the conventional electron wind force^20,21,24^. Theoretically, atoms located at the cores of lattice defects can be stimulated to vibrate around their equilibrium positions at the moment of electrical pulsing, which can overcome the Peierls barrier and facilitate dislocation motion^15,25^. Such random atom vibration is likely to induce nondirectional dislocation motion, in contrast to those induced by electron wind force. However, in most circumstances, this athermal effect often tangles with the electron wind effect^12,20^, which thus largely stays at the theoretical stage due to the lack of solid experimental evidence. Revealing the origin of electroplasticity under electropulsing remains challenging, especially at the defect level (including the defect generation, evolution, annihilation, and their interaction with interfaces).

In this study, we use Au nanocrystals as an ideal model to systematically investigate the evolution of lattice defects under electrical pulse via in situ electrical testing inside transmission electron microscopy (TEM). The Joule heat was minimized by reducing the pulse width down to 3 ns, while the defect density was reduced to the minimum to exclude the influence of possible defect interference. The generation, migration, and annihilation of defects under electropulsing were investigated dynamically. An electropulsing enhanced defect mobility has been revealed by monitoring the migration behavior of individual incoherent twin boundary (ITB), providing solid evidence of the direct electron-dislocation interaction beyond the classic model of electron wind force. These results provide insights into the origin of electrical degradation in MEMS under pulse condition, which would benefit the optimal design of micro/nano-electronic devices.

## Results

### Design of experimental protocol

**Fig. 1:**
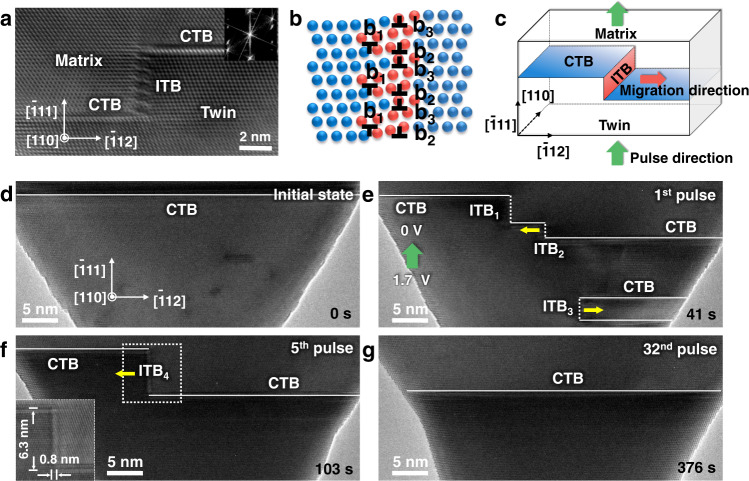
Pulse-induced migration of Σ3{112} ITB in an Au nanocrystal. **a** Atomic image of a Σ3{112} incoherent twin boundary (ITB). Inset is the Fast Fourier-filtered (FFT) image, showing a typical twin structure. **b** Atomic structure of the Σ3{112} ITB, consisting of Shockley partials *b*_1_, *b*_2_, and *b*_3_ on {111} planes. The red atoms represent the stacking faults bonded by the Shockley partials of the ITB. **c** Schematic of the experimental setup. **d**–**g** Generation, migration, and annihilation of ITBs in an Au nanocrystal under the sequentially-applied individual pulse of (1.7 V, 3 ns). CTBs (coherent twin boundaries) and ITBs are marked by the white solid and dash lines, respectively. The green arrow in **e** indicates the direction of the conventional electrical current, opposite to the direction of drift electrons. The yellow arrows in **e** and **f** represent the migration directions of ITBs.

To decouple the pulse-induced electroplasticity from electron wind force, as well as quantify the dislocation activities, the motion of dislocations should be constrained on a specific slip plane. Under electropulsing, different kinds of lattice defects, including dislocations, nanotwins, ITB, stacking fault tetrahedra, and 9R phases, were observed in face-centered cubic Au metallic nanocrystals (Supplementary Fig. 1). Among these defects, Σ3{112} ITB provides an idea platform to study the dislocation activities under electropulsing. As shown in Fig. 1a, b, a Σ3{112} ITB can be considered as a dislocation wall consisting of Shockley partials (*b*_1_, *b*_2_, and *b*_3_ = 1/6<112>)^26–28^. The slip of partial dislocations in ITB is strongly confined on a specific {111} plane parallel to the coherent twin boundary (CTB), such that the slip plane of partial dislocations is irrelevant to the direction of external loading and the dislocation cross-slip can be effectively suppressed, as schematically shown in Fig. 1c. With this idea platform, the contribution of electron wind force on electroplasticity^19,20^ can be decoupled and quantified, by monitoring the ITB migration under in situ electropulsing. Here, we delicately applied the electropulsing perpendicular to the ITB under high resolution TEM (HRTEM), where the effect of electron wind force on directional dislocation motion can be largely eliminated due to the absence of resolved shear stress on their slip planes (Fig. 1c).

Figure 1d–g show an example of defect dynamics under pulse condition. Before electropulsing, an Au nanocrystal with a Σ3{111} CTB was fabricated in situ inside a TEM (see “Methods”). CTBs are marked by the white solid lines and imaged along [110] zone axis (Fig. 1d–g). Then, an electrical pulse of (1.7 V, 3 ns) was applied perpendicular to the twin plane (along the axial direction of the nanocrystal), as shown in Fig. 1e. After the first electropulsing, the structure of CTB was destroyed, along with the generation of some ITBs, see ITB_1_, ITB_2_, and ITB_3_ in Fig. 1e. After four additional, identical pulses (the 5th in the pulse sequence), ITB_2_ migrated leftward continuously and merged with the ITB_1_, forming a new ITB_4_ with a compact core structure (6.3 nm in height and 0.8 nm in width, see the Inset in Fig. 1f). In the meantime, the ITB_3_ migrated and annihilated to the free surface (Fig. 1g). The ITB_4_ also moved and annihilated to the left free surface after the 32nd pulses, associated with the downward migration of the whole CTB. These observations indicate that the electropulsing can promote the mobility of lattice defects in metals, consistent with previous observations in literatures^22,29^. However, given that the slip of dislocations in ITB was confined to the planes perpendicular to the electron wind, dislocation motion observed here should not be directly relevant to the electron wind force, well illustrating the design of our experimental protocol.

### Migration of ITB under electropulsing

**Fig. 2:**
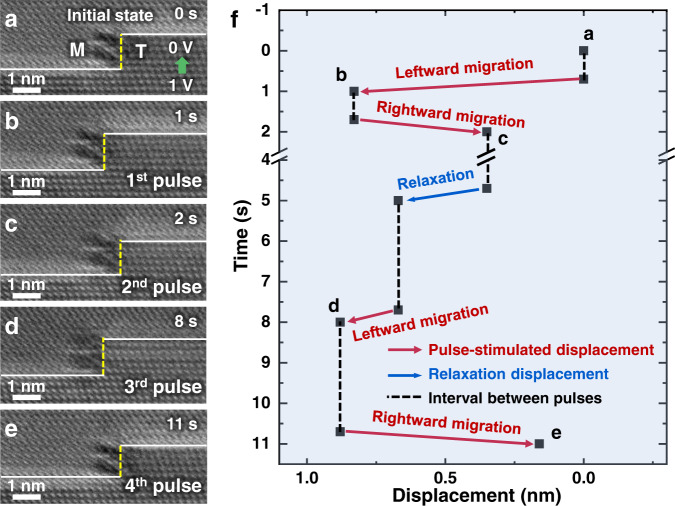
Non-directional migration of a Σ3{112} ITB under electrical pulses. **a** A Σ3{112} ITB in another Au nanocrystal. Pulses of (1.0 V, 3 ns) were sequentially applied perpendicularly to the CTB. M and T represent the matrix and twin in Au nanocrystal, respectively. The green arrow indicates the direction of the electrical current. **b**–**e** Dynamic snapshots showing the non-directional migration of the ITB under electrical pulses. **f** Migration displacements of the ITB. The red and blue arrows represent the migration distance and direction under electropulsing and relaxation, respectively. The black dash lines represent the intervals between pulses.

To quantify the relation between ITB migration and electropulsing, mechanical response of ITB stimulated by individual pulse was carefully investigated in additional examples. Figure 2a presents a magnified image of an Σ3{112} ITB in another sample (Supplementary Fig. 2 presents the overall morphology of this sample). During this experiment, pulses of (1.0 V, 3 ns) were applied perpendicular to the ITB (same as the setup in Fig. 1). Snapshots in Fig. 2b–e show the migration behavior of this ITB during the sequentially-applied pulses (see Supplementary Movie 1). Clearly, this ITB migrated only at the moment of each electropulsing, indicating that the migration of ITB was induced by electropulsing; however, the migration direction of this ITB exhibited a random behavior during the consecutive electropulsing, as shown by the pulse-stimulated alternative leftward/rightward displacements in Fig. 2b–e. Figure 2f quantitatively plots the migration direction and displacement of this ITB with respect to the experimental time. Notably, the ITB migration was directly stimulated by the application of individual pulse, and showed a non-directional behavior. Since the direction of electron wind force was perpendicularly to the slip plane of partial dislocations in the ITB, it would result in a zero (or near-zero) Schmid factor, and therefore, the electron wind force should play little role in the pulse-simulated ITB migration. We also noticed that a spontaneous relaxation occurred during the interval between the 2nd and 3rd pulses, which was likely induced by the stored residual stress. These observations suggest that there should be other factors of electron-dislocation interaction controlling the ITB migration during electropulsing, beyond the classic model of electron wind force^21,29^.

**Fig. 3:**
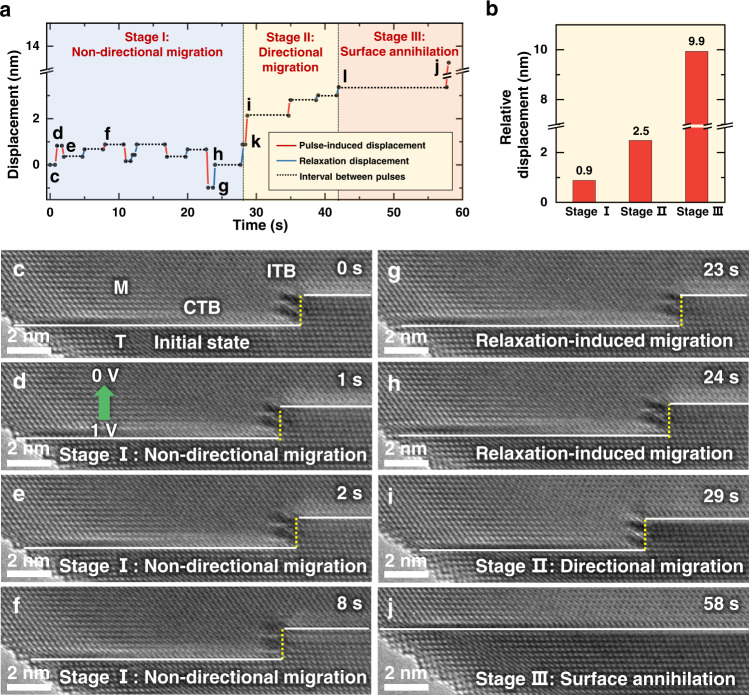
Migration and annihilation of a Σ3{112} ITB under pulses. **a** Migration displacements of the ITB with time. The migration displacements during electropulsing and relaxation are marked by red and blue solid lines, respectively. The black dash lines show the intervals between each migration. **b** Relative displacements during the three stages, with respect to the initial ITB position of each stage. **c**–**f** Non-directional migration of ITB under the pulses of (1.0 V, 3 ns) in stage I. Σ3{112} ITB and CTBs are marked by the white dash lines and yellow dash lines, respectively. M and T represent the matrix and twin in Au nanocrystal, respectively. The green arrow in **d** indicates the direction of the electrical current. **g**, **h** Relaxation-induced spontaneous ITB migration after electropulsing. **i** Directional migration induced by electropulsing during stage II, where the image force became dominated. **j** Fast surface annihilation after a pulse (stage III).

With the nanosized sample, surface image force can also influence the defect dynamics, however, it tends to cause a directional defect migration toward surface and results in the defect annihilation at surface^30^. In our experiments, the pulse-stimulated ITB migration and its random behavior in multiple electropulsing can largely exclude the possible influence of surface image force. To further clarify their difference, we applied the electrical pulses continuously until the surface annihilation of ITB, as shown in Fig. 3. Figure 3a quantifies the migration distance and direction of the ITB under each pulse. Interestingly, two different types of ITB migration can be identified during the whole process, i.e., the pulse-stimulated and the relaxation-induced ones; while based on the migration behaviors, the whole process can be classified into three different stages, including non-directional migration, directional migration toward surface and fast surface annihilation. Specifically, stage I was dominated by the randomly-occurred non-directional ITB migration, where the ITB migration was mainly stimulated at the moment of pulse application and typically showed a non-directional behavior (Fig. 3c–f). Although minor relaxation process also occurred during stage I (Fig. 3g, h), the overall process was dominated by the pulse-stimulated ITB migration. Stage II was characterized by the directional migration of ITB toward the left surface (Fig. 3i), during which the image force became to dominate the process. As shown in Fig. 3a, during the stage II, both the relaxation-induced and pulse-stimulated ITB migration tended to proceed directionally toward the nearby surface (leftward) in a stop-and-go manner. Above comparison further indicates the increasing contribution of surface image force on the directional migration of ITB during the stage II, which, as the ITB moved to a near surface position, can easily induce a fast annihilation under pulse, i.e., the stage III (Fig. 3j). Figure 3b shows the relative displacements during these three stages, with respect to the initial ITB position of each stage. The randomly-occurred non-directional ITB migration induced a small relative displacement (merely 0.9 nm) in stage I; while, the directional ITB migration toward surface resulted in large relative displacements in stages II and III, indicating the important contribution of surface image force during these stages. Note that, in contrast to the instant migration at the moment of electropulsing, the relaxation-induced ITB migration often occurred over a few seconds after electropulsing (Fig. 3g, h), which thus should be governed by the competition between the surface image force, the local residual stress generated in previous electropulsing process and the lattice resistance, as discussed later.

### Mechanism of pulse-induced electroplasticity

Theoretically, electroplasticity can be explained by the thermally-activated dislocation dynamics^12,20,25^, as described by Arrhenius equation^20,22^:

1 $$\dot{\gamma }={\dot{\gamma }}_{0}\,{\exp }\left[-\frac{\varDelta H\left(\tau \right)}{{kT}}\right]$$

where $\dot{\gamma }$ is the strain rate, ${\dot{\gamma }}_{0}$ is a pre-exponential factor which includes the density of mobile dislocations, the vibration frequency of dislocation and some other parameters, as discussed later. $\varDelta H\left(\tau \right)$ is the activation energy, $T$ is the temperature and $k$ is the Boltzmann constant. Although the electron wind was believed to increase the effective shear stress and thus decrease $\varDelta H\left(\tau \right)$^31^, its contribution to electroplasticity remains under debate due to the interference of high density of defects and the absence of direct experimental observation interlinking the dislocation motion and electron wind force^7^. Here, the dedicatedly-designed experiments in our study effectively decoupled the electron wind force and thus largely eliminated its effect on defect dynamics.

Under electrical current, the unavoidable Joule heat can soften materials by reducing the lattice resistance and activating the dislocation motion^22^. In classic theory, the Joule heat, associated thermal stress and thermal gradient serve as the major thermal effects for electroplasticity^22,32^. In our experiments, however, the thermal effects of nanosecond electrical pulse should be negligible. Firstly, the temperature rise under the ultrashort pulses are estimated to be: 29–42 °C for the pulse of (1.0 V, 3 ns) and 83–120 °C for the pulse of (1.7 V, 3 ns) (see details in Supplementary Discussion 1.1). Such temperature rises, in conjunction with the nanosecond pulse width, should have negligible contribution to activate the dislocation motion, compared with the temperature rise of several hundred degrees with long-time exposure reported in other works^8,33^. Secondly, with the high thermal conductivity (about 318 W m^−1^ K^−1^ for Au^34^), huge heat dissipator (millimeter-size Au substrate at the two ends), and nanosecond electrical pulse (merely 3 ns), the Joule heat can be rapidly dissipated^35^ (within 7.9 × 10^−11^ s by estimation, see details in Supplementary Discussion 1.2). This dissipation time of thermal heat is much smaller than the time for a single migration of ITB. Thus, the thermal effects of electrical pulse should have negligible contribution to the ITB migration observed here, further suggesting that the ITB migration should be induced by an athermal effect of direct electron-current interaction.

**Fig. 4:**
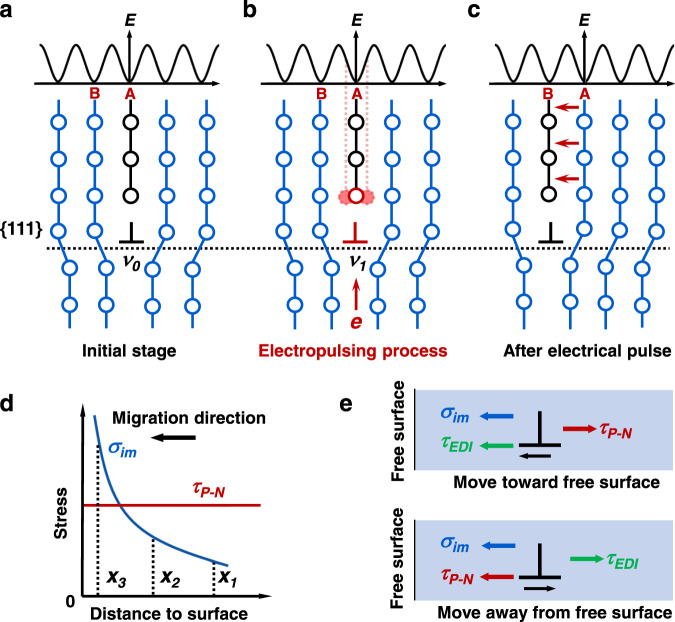
Schematic showing the direct electron-dislocation interaction during electropulsing. **a** A lattice dislocation at an equilibrium position with the lowest energy state. **b** The vibration frequency of atoms is enhanced due to the energy input by electropulsing. The activated core atoms are shown by the red solid circles, which frequently deviate from their equilibrium positions. **c** Promoted dislocation motion (from position A to the nearby position B) over the Peierls barrier. **d** Relative magnitudes of the stress induced by surface image force (*σ*_im_) and the Peierls–Nabarro stress (*τ*_P–__N_), with respect to the distance of ITB to the free surface. *x*_*1*_, *x*_*2*_*,* and *x*_*3*_ correspond to the relative positions of ITB in stages I, II, and III in Fig. 3. **e** Variation of stress states as dislocation migrates toward different directions.

As suggested by theoretical studies^12,36^, the direct electron-current interaction can influence the pre-exponential factor ${\dot{\gamma }}_{0}$ in Eq. (1), which may be the possible origin of enhanced defect mobility during electroplasticity. Since the parameters like mobile dislocation density and area of the slip plane swept out per successful fluctuation (which can influence the pre-exponential factor ${\dot{\gamma }}_{0}$) did not change significantly in our experiments (see details in Supplementary Discussion 2), the vibration frequency ($\nu$) of dislocation should be the most important parameter determining the value of ${\dot{\gamma }}_{0}$. During electropulsing, moving electrons can interact strongly with the external electric field associated with the electrical pulse. Theoretical studies have shown that the distortion of electronic structure is conducive to the formation of defects in the crystal^37^, thereby promoting the dislocation motion via enhanced vibration frequency of atoms^20,24^. Since this lattice vibration is a localized phenomenon irrelevant to the Joule heating directly, it should be regarded as an athermal effect. As schematically shown in Fig. 4, atoms in partial dislocations of ITB possess an equilibrium vibration frequency (${\nu }_{0}$) before the electropulsing. Upon electropulsing, drifting electrons with high-energy interact extensively with the atoms allocated at the dislocation core, stimulating them toward a higher vibration frequency (${\nu }_{1}$). With the enhanced vibration frequency, atoms at the dislocation core are endowed with higher possibility to relocate to the nearest positions with higher energy. Consequently, the electron-activated dislocation can overcome its Peierls barrier much more frequently. Eventually, dislocation moves instantly from its initial equilibrium position A to a new equilibrium position B at the nearby site (Fig. 4c). This process is consistent with our observations of dislocation migration in ITB only at the moment of electropulsing, where dislocations merely slipped limited distance during each pulse (Fig. 2). Moreover, this type of dislocation migration is non-directional and can happen in any equivalent slip directions, differing from the directional migration driven by the electron wind force^7,21^.

To quantify the origin of electroplasticity, stresses induced by electron-dislocation interaction were quantitatively calculated. As shown in Fig. 3, pulse-induced dislocation motion typically exhibited a random direction (stage I), i.e., independent on the direction of electron wind. In nanocrystals, the Peach–Koehler stress of a partial dislocation under the equilibrium state (before pulsing) should consist of the stress induced by surface image force (*σ*_im_, acting as the driving force) and the Peierls–Nabarro stress (*τ*_P–N_, acting as the resistance). As the ITB moves toward the free surface, the Peierls–Nabarro stress remains constant but the stress induced by surface image force increases sharply^38^ (Fig. 4d). At the moment of electropulsing, dislocations can move toward any neighboring equilibrium position due to the activation from electron-dislocation interaction (see Fig. 4a–c). This electron-dislocation interaction will generate a non-directional stress on dislocation (denoted as *τ*_EDI_), which should be a constant value if the current density remains unchanged. Careful analysis shows that the value of *τ*_EDI_ in our case is on the order of 5.1 × 10^−2^–7.4 × 10^−2^ GPa (see details in Supplementary Discussion 3). Meanwhile, the magnitude of stress induced by electron wind force (*τ*_ew_) can be calculated by^22^:

2 $$ {\tau }_{{{{{{{\rm{ew}}}}}}}}\,\approx \,\frac{{m}_{e}{\nu }_{\tiny F}j}{3{{{{{\rm{\it{e}}}}}}}}$$

where ${m}_{e}$, $j$, ${\nu }_{\tiny F}$, and $e$ are the electron mass, pulsed current density, Fermi velocity, and electron charge, respectively. Given the maximum current density of ~ 1.7 × 10^9^ A cm^−2^ during pulsing (see Supplementary Discussion 4 for current estimation), the maximum stress induced by electron wind force is estimated to be ~ 0.9 × 10^−2^ GPa, which is about eight times lower than that of *τ*_EDI_. Thus, the ITB migration observed in this study should be likely stimulated by the electron-dislocation interaction (due to the enhanced vibration frequency $\nu$, as discussed above), rather than the electron wind force. It needs to note that the stress state for dislocation motion varies with its distance to surface (Fig. 4d) and its migration direction (Fig. 4e). When the ITB was far away from free surface, *σ*_im_ was relatively small (*x*_*1*_ in Fig. 4d) and the main resistance of dislocation motion (along either direction) came from the Peierls–Nabarro stress *τ*_P–N_ (stage I in Fig. 3). As ITB approached the free surface, σ_im_ increased sharply such that *τ*_EDI_ cannot withstand σ_im_ (*x*_*2*_ in Fig. 4d) and *τ*_P–N_, resulting in a directional migration toward surface during the stage II. Near the free surface, *σ*_im_ should be larger than *τ*_P–N_ (*x*_*3*_ in Fig. 4d), which would induce a fast annihilation of ITB at free surface (stage III). Additionally, with the ultrashort pulse width, electropulsing-induced atom vibration cannot be instantly released after pulse, such that part of energy can be stored by the lattice distortion near the dislocation cores. During the pulse intervals, the stored energy can work with the surface image force and induce the spontaneous relaxation of ITB (Fig. 3g, h), to reduce the system energy. Such kind of ITB relaxation, to some extent, further suggests that the ITB migration was originated from the direct electron-dislocation interaction that enhanced the vibration frequency of atoms at dislocation cores, rather than the stress induced by electron wind force.

## Discussion

**Fig. 5:**
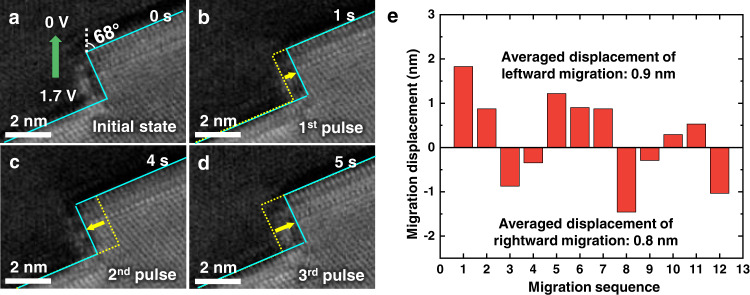
Pulsing-induced migration of an inclined ITB. **a** Initial Σ3{112} ITB. The angle between the current direction and the (111) slip plane is 68°. The green arrow indicates the direction of the electrical current. **b**–**d** Sequential snapshots showing the non-directional migration of ITB under pulses of (1.7 V, 3 ns). The positions of ITB before and after the pulses are marked by the yellow dashed lines and cyan solid lines, respectively. The yellow arrows represent the migration directions of ITBs. **e** Quantitative measurements of migration displacements under pulses. The positive and negative migration displacements represent the leftward and rightward migrations, respectively.

Both our experimental observations and theoretical analyses have demonstrated that the electroplasticity of metallic materials should be dominated by the electron-dislocation interaction, beyond the classic model of electron wind force^21,39^. In Figs. 2 and 3, partial dislocations in ITB were confined to the slip planes perpendicular to the motion direction of electrons, where the Schmid factor on the slip plane is zero. To further validate the generality of our conclusion, an inclined ITB was fabricated and investigated to see its migration behavior under electropulsing (Fig. 5). Supplementary Fig. 2b shows the overall morphology of this sample, where the angle between the motion direction of drift electrons and the (111) slip plane is 68°. This inclined setup can induce a large shear component of the electron wind force on the slip plane of ITB. However, under electropulsing, the ITB still showed a non-directional migration behavior (Fig. 5a–d). This phenomenon validates again that the electron wind force should have less contribution to the migration of lattice defects, supporting our conclusion that the electroplasticity of metallic materials is dominated by the direct electron-dislocation interaction that significantly enhances the atom vibration at dislocation cores. Quantitative measurements in Fig. 5e further show that although the electron wind force is non-decisive factor to stimulate the electroplasticity, it may benefit the defect migration to some extent. As shown in Fig. 5e, the frequency and averaged displacement of leftward migration are slightly higher than that of the rightward migration, because the electron wind force can exert a shear stress toward the bottom left direction under this condition, benefiting the leftward migration. In the cases of samples with larger dimensions (e.g., micro- or sub-micro- scale^7,21,29^), dislocation can slip along certain direction of majority carriers. This is not in conflict with our observations, since, from a statistical view, the possibility of defect migration under drift electrons is more favorable in dynamics and energetics, as indicated by Fig. 5e. While in our properly-designed experiments at atomic level, the contribution of direct electron-dislocation interaction that enhances the atom vibration at dislocation cores was revealed by decoupling the electron wind effect and reducing the defect density to a minimum level, providing new insights into the origin of electroplasticity, in contrast to the driving force of electron wind force in classic model^7^.

Besides the direct electron-dislocation interaction and stress induced by electron wind force, there are some other factors that may influence the migration of ITB in our experiments. Firstly, the current-induced electromigration can generate a vacancy concentration gradient due to the atoms flux induced by electromigration. This vacancy concentration gradient can lead to the atomic diffusion, which moves against the electromigration flux and thus induces a back-stress that may influence the dislocation motion^1,40–42^. However, the occurrence of electromigration needs dozens of hours and the resultant back-stress needs enough time to build up (for the accumulation of vacancy gradient)^40^; while, with the nanosecond pulse in our experiments, the contribution of electromigration and resultant back-stress should be negligible. It is also noticed that the transient second density-changing effect created by the electron wind can induce a pressure gradient that increases linearly with the distance along the axial direction of sample^43^. But in our experiments, the non-directional migration of ITB mainly proceeded along the radial direction of our samples, and thus the pressure resulted from the electron wind force should be constant or have very limit variation during the ITB migration, which should not influence the conclusion of our experiments. Secondly, the skin effect, referring to the localization or concentration of current near a specimen surface^44^, may influence the stress magnitude of electron-dislocation interaction near free surface. Within the nanosecond pulse width, the skin depth *δ* in our sample is calculated to be ~ 1.6 × 10^−5^ m (see details in Supplementary Discussion 5), which is about three orders larger than the diameters of our specimens (typically less than 20 nm, on the order of 10^−8^ m). Therefore, the current should be uniformly distributed throughout the specimens in our experiment, with less contribution of skin effect. Besides, we also noticed that the magnitude of current density can influence the migration distance. As shown in Supplementary Fig. 3, the lower part of ITB with smaller cross-sectional area migrated first while the upper part remained at its original position owning to a higher current density, which indicates that the stress magnitude of direct electron-dislocation interaction is closely related to the current density. Last, although the non-directional migration of ITB provides solid evidence that direct electron-dislocation interaction controls the electroplasticity in metallic materials beyond the electron wind force, more experiments are required to further study this phenomenon across different length scales for a systematical understanding.

In summary, we reveal the mechanism of electroplasticity in metallic materials, by decoupling the contribution of electron wind force via the properly-designed in situ TEM electropulsing experiments. Σ3{112} ITBs can be stimulated to migrate upon electropulsing, which exhibited a non-directional migration behavior independent of the electron direction, in contrast to the widely-believed directional migration under electric filed. Quantitative analyses demonstrate that the non-directional ITB migration was governed by the electron-dislocation interaction that enhanced the atom vibration at dislocation cores, rather than the electron wind force. These findings shed new insights on the origin of electroplasticity in metals and alloys at atomic level, which are of scientific and technological significances for understanding the electromigration and resultant electrical damage/failure in a broad class of micro-/nano-electronic devices, especially the ones with tens of nanometer size.

## Methods

### In situ nanofabrication and electropulsing experiment

The in situ electrical experiments were carried out using a PicoFemto® TEM electromechanical holder from Zeptool Co. inside a FEI Titan G^2^ 60–300 TEM (operated at 300 kV). Before electropulsing, an Au nanocrystal was made via an in situ welding method. Firstly, Au rod (99.99 wt.%, Alfa Aesar Inc.) with 0.25 mm in diameter was cut by a wire cutter to get clean surface with numerous nanoscale tips. Then, the Au rod was loaded onto the static side of TEM holder, while a needle-like Au rod was used as a scanning tunneling microscope (STM) probe on the other side of the TEM holder (Supplementary Fig. 4a). Before each experiment, we searched on the fracture surface of the fractured Au rod to find a nanoscale tip with the zone axis of [110] to enable the atomic resolution for in situ testing. Then, the needle-like Au probe was driven by a piezo-controller to make a point contact with the nanoscale tip in [110] zone (Supplementary Fig. 4b, c). A Keysight pulse generator (81110A, configured with one output module “Agilent 81111A 165 MHz 10 V”) was used to apply a square electrical pulse on the point contact, which, with the instantaneous high-density energy at the moment of pulse, can weld the two nanotips together, forming a nanocrystal with multiple TBs^14,35,45^ (Supplementary Fig. 4d). Finally, individual pulse of (1.0–1.7 V, 3 ns) was sequentially applied on the nanocrystal, leading to the defect generation, migration, and annihilation. No external mechanical loading was applied during the electropulsing or pre-applied before the electropulsing in all experiments. During experiments, a Gatan 994 charge-coupled device (CCD) camera was used to record the defect dynamics in real time at a rate of ~0.3 s per frame. Note that the resistive-capacitive (RC) effects resulted from the stray capacitance in the circuits might lengthen the pulse width. Through careful calculations (see details in Supplementary Discussion 6), it is concluded that the effects arise from the longer pulse width (thermal dissipation and skin effect) should be negligible in our experiments. Thus, the variation in the pulse width is insignificant on the conclusion of our experiments.

### Measurements of resistance of Au nanocrystals

Resistances of Au nanocrystals were measured to quantify the current density under electropulsing. Considering the intrinsic experimental error in two-wire resistance sensing, the contrast experiment was carried out to measure the resistance of Au nanocrystal. A source meter instrument (Keithley 2611B SYSTEM Source Meter®) was used as a constant current output. Firstly, the resistances of the circuit with an Au wire (0.25 mm in diameter) were measured several times under different conditions, including before the application of pulses, during the intervals of pulses, and after the application of several pulses (Supplementary Fig. 5a). Then, the resistances of the circuit with Au nanocrystal were measured in the same way (Supplementary Fig. 5b). As shown in Supplementary Fig. 5c, the resistances in these two sets of measurements are ranging in 9.7–10.1 Ω (Au wire) and 73.1–77.7 Ω (Au nanocrystal), respectively, with small variations between pulses. Thus, the resistance of Au nanocrystals is ranging in 63.0–67.3 Ω, with the resistivity of ~20 μΩ cm (sample size for estimation: 100 nm in height and 10 nm in average radius; see details in Supplementary Discussion 4). This value is in consistent with results reported in previous study of Au nanostructures^46^, verifying the validity of the measurements.

## Supplementary information

Supplementary Information

Description of Additional Supplementary Files

Supplementary Movie 1

## Data Availability

The data that support the findings of this study are presented in the paper and the Supplementary Information.
