# Supplementary material for: Revealing the pulse-induced electroplasticity by decoupling electron wind force
Source: Nat Commun. 2022 Oct 31;13:6503. doi: 10.1038/s41467-022-34333-2 (PMC9622885; doi:10.1038/s41467-022-34333-2)
Supplement: Supplementary file 1 — Supplementary Information [file 41467_2022_34333_MOESM1_ESM.pdf]

Supplementary Information for

Revealing the pulse-induced electroplasticity by decoupling electron wind force

Xing Li, Qi Zhu, Youran Hong, He Zheng, Jian Wang, Jiangwei Wang*, Ze Zhang*

<u>This supplementary information includes:</u>

Supplementary Figures 1-5

Supplementary Discussions 1-6

Supplementary References

Supplementary Figures

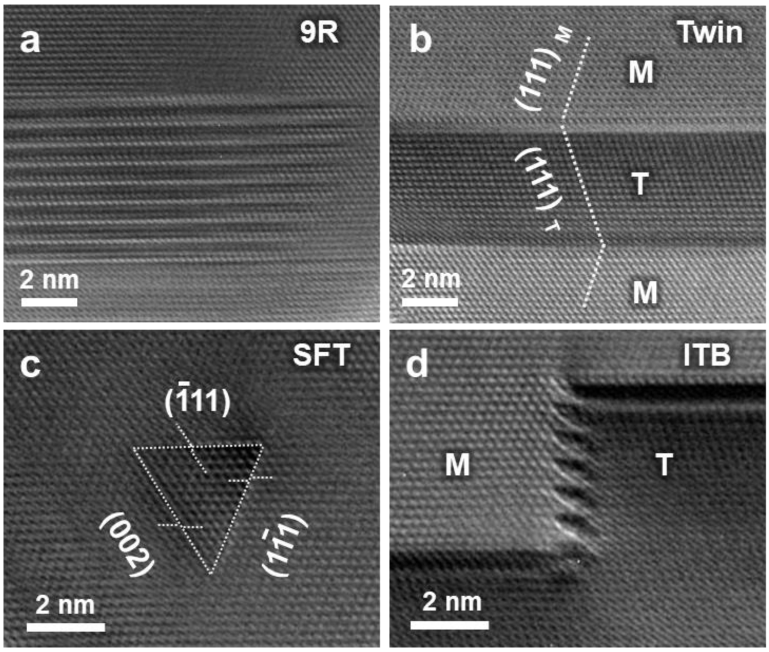

Supplementary Figure 1 Different defects induced by electrical pulses. (a) 9R phase. **(b)** Twins. **(c)** Stacking fault tetrahedra (SFT). **(d)** Σ3{112} inherent twin boundary (ITB).

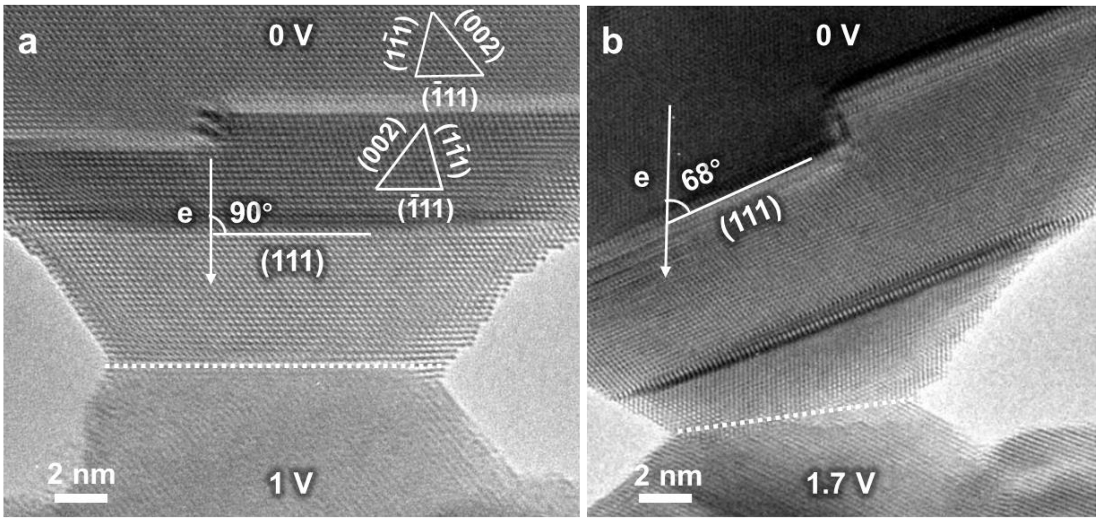

Supplementary Figure 2 Initial morphologies of two Au nanocrystals. (a) Morphology of the Au nanocrystal presented in Fig. 2, viewed along [110] zone axis. The grain boundary is marked by the white dash line. The direction of pulses (1.0 V, 3 ns) is perpendicular to the twin boundary (TB), as shown in Fig. 2. **(b)** The morphology of the Au nanocrystal with an inclined TB presented in Fig. 5. The angle between the motion direction of drift electrons and the (111) slip plane is 68°. The white arrows indicate the motion direction of the drift electrons.

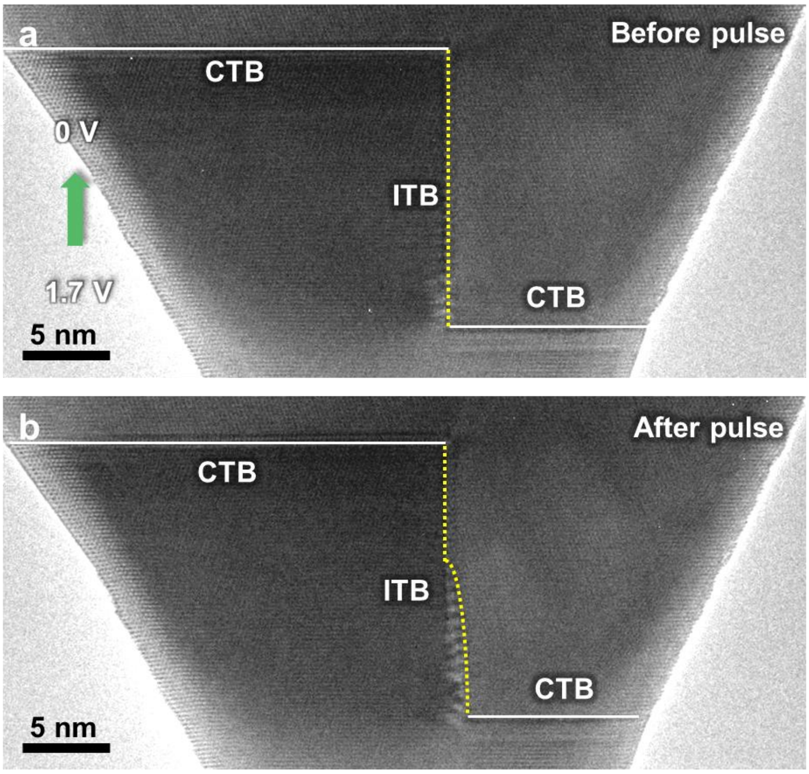

Supplementary Figure 3 *In situ* snapshots of an Au nanocrystal with a relatively large ITB before (a) and after (b) an electrical pulse of (1.7 V, 3 ns). The lower part of the ITB migrated while the upper part remained at its original position. The green arrow in (a) indicates the direction of the electrical current.

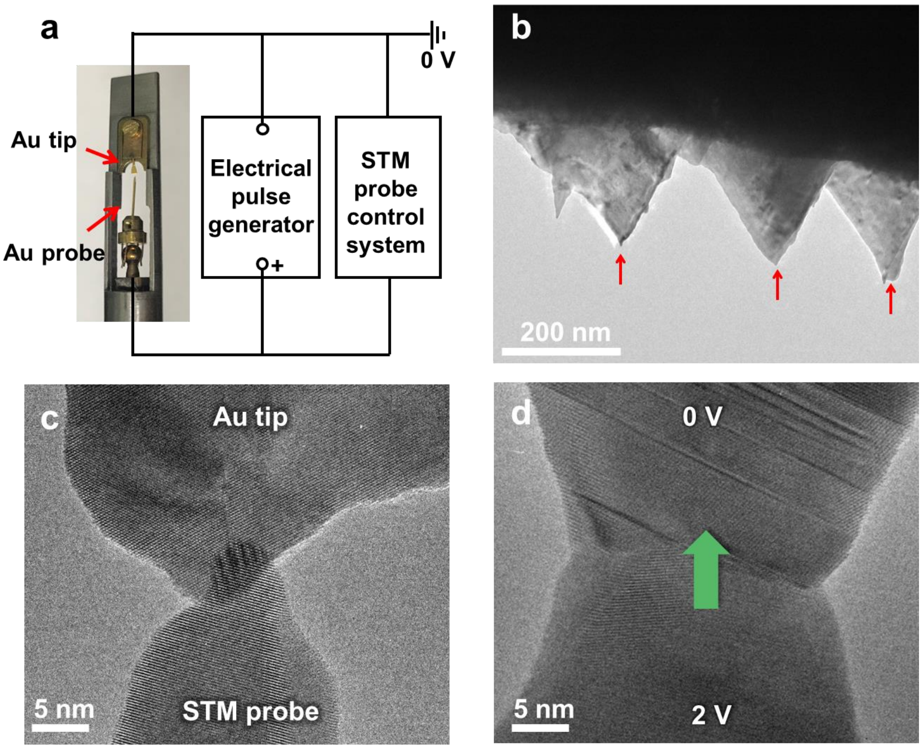

Supplementary Figure 4 Experimental setup of *in situ* electropulsing test. (a) The transmission electron microscopy (TEM)-scanning tunneling microscope (STM) platform for *in situ* electrical pulsing experiment. **(b)** The fractured Au rod with numerous nanoscale tips. **(c)** The Au probe was driven to contact with a nanoscale tip on the opposite side. **(d)** After a short electrical pulse on the point contact, a nanoscale crystal containing multiple TBs can be fabricated. The green arrow indicates the direction of the electrical current.

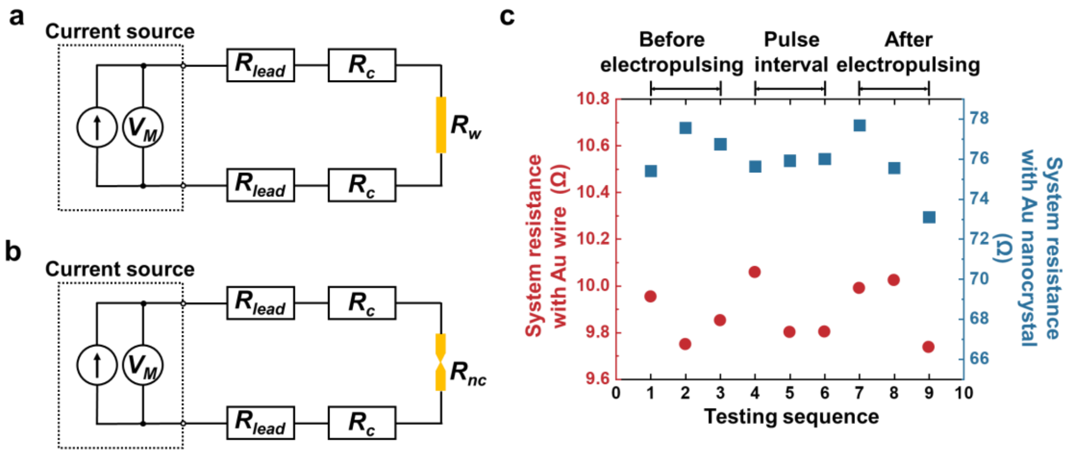

Supplementary Figure 5 Measurements of the resistance of Au nanocrystals. (a) Resistance measurement of the circuit with bulk Au wire. **(b)** Resistance measurement of the circuit with Au nanocrystal. V_M is the measured voltage of the external circuit. R_W is the resistance of Au wire, R_{nc} is the resistance of Au nanocrystal, R_{lead} and R_c is the lead resistance and contact resistance, respectively. **(c)** The electrical resistance measured under different conditions, including before the application of pulses, during the intervals of pulses and after the application of several pulses. The red circles represent the system resistance with Au wire, and the blue squares represent the system resistance with Au nanocrystal, both of which show small variations in our experiments.

Supplementary Discussions

1. Evaluation of thermal effects

Under electrical current, the unavoidable Joule heat may soften materials by reducing the lattice resistance and activating the dislocation motion[1]. In classic theory, the Joule heat, associated thermal stress and thermal gradient serve as the major thermal effects for electroplasticity[1,2]. In our experiments, however, the thermal effects of nanosecond electrical pulse should be negligible, as discussed below.

1.1 Estimation of the temperature rise during the nanosecond pulse

The temperature rise induced by the electrical pulse in bulk sample can be given by[3]:

$$\varDelta T_m = \frac{U^2 t}{R C_p m} \tag{1}$$

where U is the voltage, t is the pulse width, R is the resistance, C_p is the specific heat capacity and m is the mass of specimen. Due to the limitation of experimental setup, it is difficult to accurately measure the exact resistance of Au nanocrystal, and C_p may also deviate from the value of bulk samples[4]. Besides, with the ultrahigh thermal diffusivity (1.27×10^{-4} m^2 s^{-1}) and thermal conductivity (317.422 W m^{-1} K^{-1}) of Au matrix, the actual temperature rise in the specimen could be less than the calculated value[3]. Here, we managed to conduct an *in situ* experiment to estimate $\varDelta T_m$. Firstly, we use Au nanocrystals with the same size and morphology as the specimen in Fig. 2 of the manuscript, such that their resistance, specific heat capacity and mass are almost the same. By keeping the pulse duration to the same value (3 ns), the temperature rise $\varDelta T$ under different pulse voltage is proportional with U^2:

$$\frac{\varDelta T_1}{\varDelta T_2} = \frac{U_1^2}{U_2^2} \tag{2}$$

If we can obtain the critical pulse voltage (U_{melt}) that can melt the Au nanocrystal (the

melt point of bulk Au is 1064 °C), the temperature rise under the pulse of (1.0 V, 3 ns) can be roughly estimated by:

$$\Delta T_1 = \frac{U_1^2}{U_{melt}^2} \cdot \Delta T_{melt} \quad (3)$$

Following this idea, we conducted several controlled electropulsing experiments to melt the Au nanocrystals. Supplementary Fig. 6 shows an example. The melt voltage (U_{melt}) of the pulse was measured to be 5 ~ 6 V. With the equation (3), we can roughly obtain a temperature rise in the range of 29 °C ~ 42 °C for the pulse of (1.0 V, 3 ns) and 83 °C ~ 120 °C for the pulse of (1.7 V, 3 ns). Such temperature rises, in conjunction with the nanosecond pulse width, are too small to activate the dislocation motion, compared with the temperature rise of several hundred degrees with long-time exposure reported in other works[5,6]. Thus, the driving force of dislocation motion should mainly come from the direct electron-dislocation interaction.

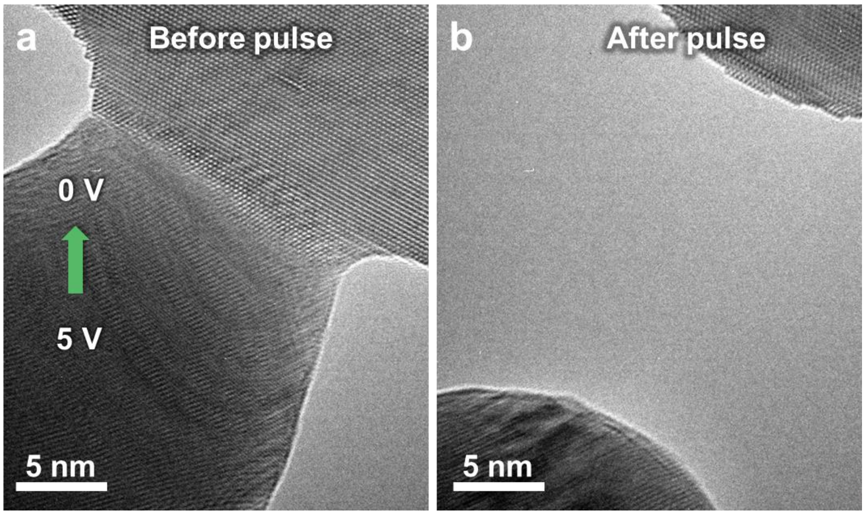

Supplementary Figure 6 A typical melting of Au nanocrystal under the critical pulse of (5.0 V, 3 ns). (a) The Au nanocrystal before applying a pulse. **(b)** The melting occurred after applying the pulse. The green arrow indicates the direction of the electrical current.

1.2 Estimation of the thermal dissipation of pulse current

The dissipation time of thermal heating is further estimated to evaluate its contribution to the ITB migration. Theoretically, the distance of thermal diffusion over time t (diffusion length l_{diff}) can be calculated by[1,7]:

$$l_{diff} \approx \sqrt{\alpha t} \tag{4}$$

where α is the thermal diffusivity of specimen and t is the time of thermal diffusion. In this equation, α can be determined by:

$$\alpha = \frac{k}{\rho c_p} \tag{5}$$

where k is the thermal conductivity, ρ is the density and c_p is the specific heat capacity. At room temperature, the typical values of these parameters for Au are[8]: k = 317.422 W m^{-1} K^{-1}, ρ = 1.932 × 10^{4} kg m^{-3}, c_p = 129 J kg^{-1} K^{-1}. Substituting these values into equation (5), the thermal diffusivity α_{Au} is estimated to be 1.27 × 10^{-4} m^{2} s^{-1}. Given the pulse width of t ~ 3 × 10^{-9} s, the diffusion length l_{diff} is estimated to be ~ 6.17 × 10^{-7} m (617 nm) by equation (4), which represents the dissipation distance of heat flux in the pulse width is 617 nm. This thermal dissipation distance is much larger than the maximum length of our Au nanocrystal (around 100 nm, see the sample morphology in the Supplementary Fig. 2). With the ultrahigh thermal diffusivity (1.27 × 10^{-4} m^{2} s^{-1}) and thermal conductivity (317.422 W m^{-1} K^{-1}) of Au, the inherent thermal gradient induced by the localized Joule heat can be readily annihilated.

Regarding the dissipation time, if we consider the length of the Au nanocrystal (~ 100 nm) as the maximum diffusion length l_{diff} in the equation (4), we can obtain a dissipation time t_d of ~ 7.9 × 10^{-11} s. This again validates that the thermal heat can be immediately dissipated. For the migration of ITB, it only occurs at the moment of electropulsing (~ 3 × 10^{-9} s). Although we cannot obtain an exact value due to the time

resolution of the charge-coupled device (CCD) camera (0.3 s per frame), based on the result in Supplementary Fig. 7, the time for a single migration of ITB should range in 3×10^{-9} s ~ 0.3 s, larger than the dissipation time of thermal heat (~ 7.9×10^{-11} s).

In summary, the thermal gradient and heat dissipation should have negligible contributions to the observed ITB migration, supporting that the ITB migration observed in our experiment should be induced by an athermal effect of direct electron-current interaction.

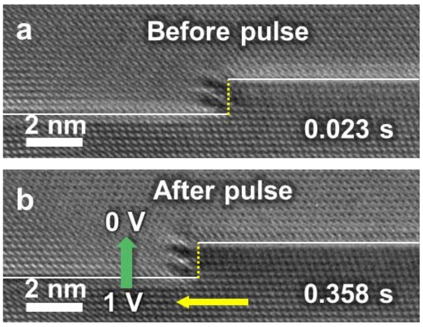

Supplementary Figure 7 Evaluation of the timescale of ITB migration. (a) The ITB kept static before applying a pulse of (1.0 V, 3 ns). The scale bar is 2 nm. **(b)** ITB stopped migration after one pulse. The green arrow indicates the direction of the electrical current, and the yellow arrow represents the migration direction of the ITB.

2. Discussion on the pre-exponential factor $\dot{\gamma}_0$

Generally, the plastic flow of materials is thermally-activated and the drift electrons can influence the pre-exponential factor $\dot{\gamma}_0$[9]:

$$\dot{\gamma}_0 = N_{d,m}(bA\nu \;/\; M)\; exp(\Delta S \;/\; k) \tag{6}$$

where $N_{d,m}$ is the mobile dislocation density, b is the Burgers vector, A is the area of slip plane swept out per successful fluctuation, ν is the vibration frequency of dislocations involved in the plastic flow, M is the Taylor orientation factor, ΔS is the entropy of activation and k is the Boltzmann constant. In our case, the dislocation structure (Σ3{112} ITB) involved in electroplastic flow did not change during the whole migration process (see Fig. 3c-j) and thus the contributions of $N_{d,m}$, b, A, and M can be ignored. ΔS is related to the temperature rise in the electropulsing process, which can be largely eliminated in our case, given the high thermal conductivity (≈318 W m^{-1} K^{-1} for Au), huge heat dissipator (millimeter-size Au substrate at the two ends), and nanosecond electrical pulse (merely 3 ns) in our experiments (see Supplementary Discussion 1). As a result, the vibration frequency ν should play an important role on the pre-exponential factor $\dot{\gamma}_0$ during the dislocation motion.

In addition, since inherent lattice vibrations or phonons are oscillations in the relative positions of atoms in the crystal lattice[10], the electron-phonon interactions at dislocation cores in the electropulsing process may introduce a disturbance into the vibrational spectrum of the mobile dislocation, thus activating the dislocation motion[11]. This enhanced vibration can be viewed as an additional lattice vibration activated beyond the normal vibration of lattice, since it is a localized phenomenon near the dislocation core, activated by the electron-dislocation interaction.

3. Quantification of the stress state

Supplementary Fig. 8 schematically illustrated the three typical migration situations (corresponding to the three stages in Fig. 3), which are discussed as follows.

Stage I: $x = x_1$, non-directional migration

When the ITB was far away from the free surface, σ_{im1} was relatively small. In Fig. 3, the distances of ITB-left surface and ITB-right surface were 13.3 nm and 18.9 nm, respectively. When applying pulsed current, the partial dislocations can move either away from or toward the free surface. This non-directional migration of ITB under electropulsing suggests that the migration of ITB needs to overcome the Peierls-Nabarro stress and the stress induced by surface image force, i.e., $\tau_{EDI} > \tau_{P-N} + \sigma_{im1}$. It needs to note that between pulses, relaxation-induced ITB migrations occurred and all proceeded toward the free surface. This implies that the residual stress induced by electropulsing, as a driving force, was too small to work against τ_{P-N} and σ_{im1}. Thus, the partials can only slip toward free surface (driven by the σ_{im1} and residual stress) without the help of electrical pulses.

Stage II: $x = x_2$, directional migration

When the ITB approached one side of the sample surface (e.g., at Point 1 in Fig. 3a, the distance between ITB and left free surface was 9.9 nm and 22.2 nm for right free surface), σ_{im} increased gradually to some value of σ_{im2}, while τ_{EDI} remained unchanged (the pulse parameters and sample geometry are the same). At this moment, $\tau_{P-N} + \sigma_{im2} > \tau_{EDI}$. As a result, the ITB cannot migrate away from free surface, and a directional migration toward left surface was exhibited.

Stage III: $x = x_3$, surface annihilation

As the ITB migrated close to the free surface, σ_{im} increased to a level higher than τ_{P-N} (denoted as σ_{im3}). Under this condition, dislocations of ITB would move

continuously and annihilate to the free surface upon electropulsing, as shown by the final migration for 9.9 nm without any stop in Fig. 3j.

According to the analysis above, we can obtain that:

$$\tau_{P-N} + \sigma_{im1} < \tau_{EDI} < \tau_{P-N} + \sigma_{im2} \tag{7}$$

Thus, by calculating the magnitudes of τ_{P-N}, σ_{im1} and σ_{im2}, the value of τ_{EDI} can be roughly estimated, as discussed following.

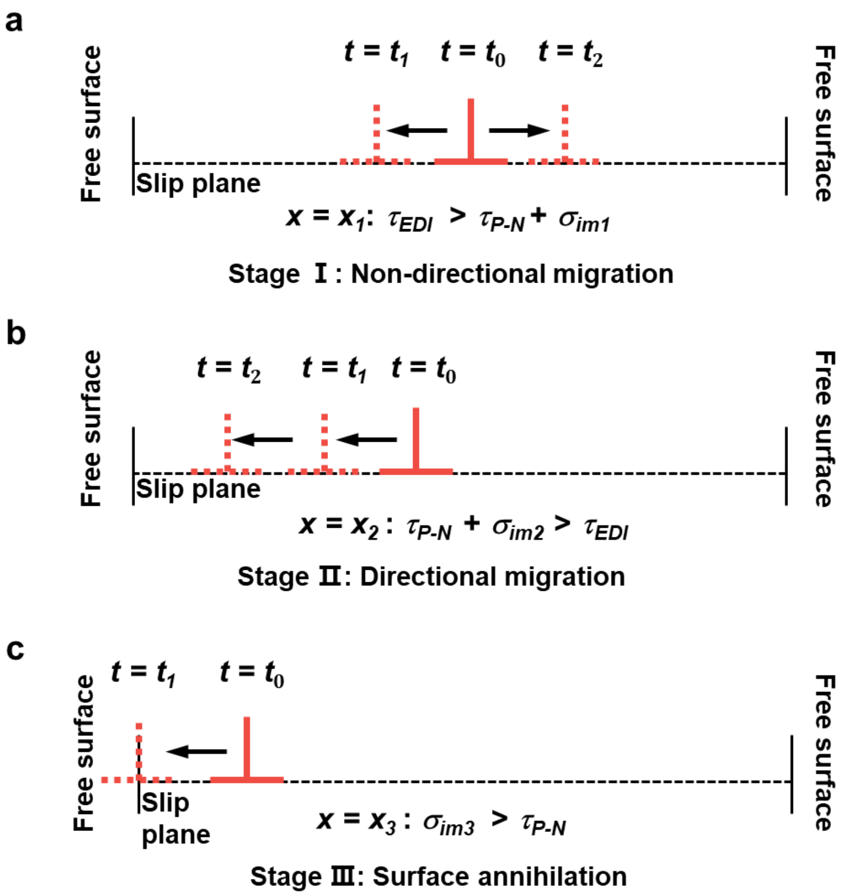

Supplementary Figure 8 Three different states as the ITB migrated toward free surface with an increasing image force. **(a)** Non-directional migration of ITB when $\tau_{EDI} > \tau_{P-N} + \sigma_{im1}$. **(b)** Directional migration of ITB when $\tau_{P-N} + \sigma_{im2} > \tau_{EDI}$. **(c)** The surface annihilation of ITB when $\sigma_{im3} > \tau_{EDI}$. Dislocations in $t = t_0$, $t = t_1$ and $t = t_2$ represent three positions in time sequence.

Firstly, we need to calculate the magnitudes of σ_{im1} and σ_{im2}. Since the ITB was deviated from the sample center toward the left surface slightly, the distances between left and right free surface must be considered at the same time when calculating the image stress. And the total image stress is[12]:

$$\sigma_{im} = \sigma_{im-left} - \sigma_{im-right} = \frac{\mu b}{4\pi(1-v)l_{left}} - \frac{\mu b}{4\pi(1-v)l_{right}} \quad (8)$$

The corresponding l_{1-left} and $l_{1-right}$ are 13.3 nm and 18.9 nm (for σ_{im1}), and l_{2-left} and $l_{2-right}$ are 9.9 nm and 22.3 nm (for σ_{im2}), respectively. The Burgers vector b of individual dislocations in ITB equals to 1/6 $[11\bar{2}]$, the Lame coefficient μ and Poisson's ratio v in the direction of {111}<112> are 26.1 GPa and 0.484 for Au[13,14], respectively. Substituting these values into the equation (8), the values of σ_{im1} and σ_{im2} are estimated to be 1.4 × 10^{-2} GPa and 3.7 × 10^{-2} GPa, respectively.

It is kind of hard to precisely calculate the magnitude of τ_{P-N}, due to the complex stress field of ITB. But in our experiments, by analyzing the migration dynamics of ITB, the value of τ_{P-N} can be estimated as follow: in stage I and II, τ_{P-N} was larger than σ_{im} because partial dislocations of ITB eventually stopped to move after the electropulsing. But in stage III, ITB migrated 9.9 nm and annihilated at free surface under the same pulse conditions in stage I and II. This unusual migration indicates that after a slight migration under pulse current, σ_{im} increased and became lager than τ_{P-N}, resulting a spontaneous migration toward surface continuously. The analyses above imply that $l = l_2$ is a transition point where $\tau_{P-N} \approx \sigma_{im2}$, corresponding to a τ_{P-N} value of ~ 3.7 × 10^{-2} GPa. Substituting the values of τ_{P-N}, σ_{im1} and σ_{im2} in equation (7), τ_{EDI} is determined to be in the range of 5.1 × 10^{-2} GPa - 7.4 × 10^{-2} GPa.

4. Measurements of electrical resistance and current density of Au nanocrystal

Resistances of Au nanocrystals were measured to quantify the current density under electropulsing. Considering the intrinsic experimental error in two-wire resistance sensing, the contrast experiment was carried out to measure the resistances of Au nanocrystals. A source meter instrument (Keithley 2611B SYSTEM Source Meter®) was used as a constant current output.

Firstly, the resistances of the circuit with an Au wire (0.25 mm in diameter) were measured several times under different conditions, including before the application of pulses, during the intervals of pulses and after the application of several pulses (Supplementary Fig. 5a). The measured resistance R_{M1} is:

$$R_{M1} = \frac{V_{M1}}{I} = R_W + 2R_{lead} + 2R_c \quad (9)$$

where V_{M1} is the measured voltage of the external circuit, R_W is the resistance of Au wire, R_{lead} and R_c is the lead resistance and contact resistance, respectively. The room temperature bulk resistivity of Au is quoted to be 2.2 μΩ cm[15] and thus the calculated resistance (R_W) of Au wire is 1.12 × 10^{-3} Ω, which is negligible. So $R_{M1} \approx 2R_{lead} + 2R_c$, which was measured to be 9.7 Ω ~ 10.1 Ω.

Then, the resistances of the circuit with Au nanocrystal were measured in the same way (Supplementary Fig. 5b), where the resistances of lead and contact remained unchanged. The measured resistance R_{M2} is:

$$R_{M2} = \frac{V_{M2}}{I} = R_{nc} + R_W + 2R_{lead} + 2R_c \quad (10)$$

where, R_{nc} is the resistance of nanocrystal. As shown in Supplementary Fig. 5c, R_{M2} was measured to be 73.1 Ω ~ 77.7 Ω. In contrast experiment, $R_W \approx 0$ Ω and $2R_{lead} + 2R_c \approx 10$ Ω. Thus, R_{nc} is estimated to be ~ 65 Ω using the equation (10), with the resistivity of ~ 20 μΩ cm (100 nm in height and 10 nm in average radius for estimation). This value is in consistent with the results reported in previous study of Au

nanostructure[15], verifying the validity of the measurements. Finally, the current density in our experiments can be calculated by:

$$J = \frac{I}{S} = \frac{U}{R_{nc}\pi r^2} \tag{11}$$

where U is the pulse voltage (1.0 V) and r is the radius of the cross section at the ITB (~ 16 nm). Given a total measured resistance of ~ 75 Ω, the current density is estimated to be ~ 1.7×10^9 A cm^{-2}.

5. Evaluation of the skin effect

The skin effect, referring to the localization or concentration of current near a specimen surface[16], may influence the stress magnitude of electron-dislocation interaction near free surface.

To evaluating the skin effect in our experiments, we have measured the output of the pulse generator using an oscilloscope, as shown in Supplementary Fig. 9. When we applied a pulse of (1.0 V, 3 ns), the measured pulse was (0.93 V, 2.83 ns) as shown by point D and time duration between B and F in Supplementary Fig. 9, matching well with the parameters we set, though an acceptable error did exist.

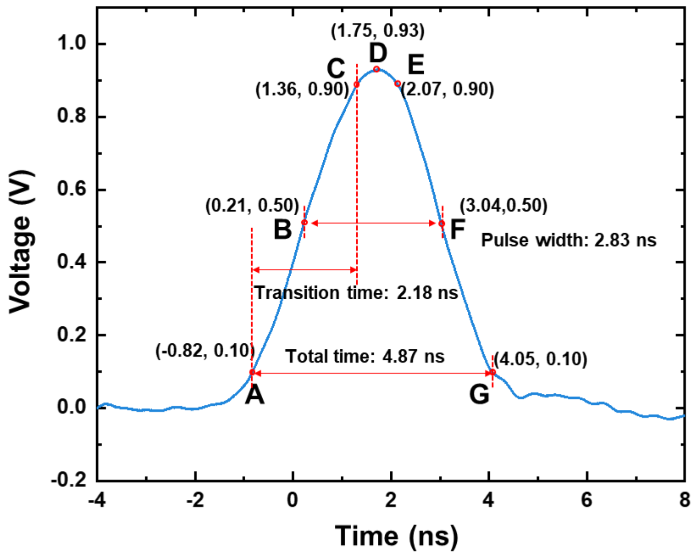

Supplementary Figure 9 The measured waveform of a pulse current. The pulse parameter was set to as (1.0 V, 3 ns), with 2 ns in transition time (interval between the 10%- and 90%- amplitude points on the leading/trailing edge, 2 ns is the minimum value for our equipment). The pulse width is 2.83 ns, defined as interval between leading- and trailing-edge medians, and the total time (start point of leading edge and the end point of the trailing edge) of tested pulse is 4.87 ns. The measured waveform matches well with the pre-set parameters with acceptable error. The output of the pulse generator was measured using an oscilloscope (Agilent, DSO9404A) which support an accuracy to two decimal places for voltage and time.

For the possible skin effect in our experiments, the skin depth δ can be calculated by[2]:

$$\delta = \left(\pi f \mu \frac{1}{\rho}\right)^{-\frac{1}{2}} = \left(\pi \frac{1}{T} \mu \frac{1}{\rho}\right)^{-\frac{1}{2}} \quad (12)$$

where f is the frequency of the pulse (which is 200MHz based on the period given below), μ is the permeability, ρ is the resistivity of the specimen and T is the period of the pulse. For one pulse applied in our experiment, $T \approx 5$ ns (see Supplementary Fig. 9), $\mu = 4\pi \times 10^{-7}$ H m^{-1}, $\rho \approx 20$ μΩ cm (Supplementary Discussion 4). Substituting these values into equation (12), we obtain a skin depth δ of ~ 1.6×10^{-5} m. This value is about three orders larger than the diameters of our specimens (typically less than 20 nm, on the order of 10^{-8} m). Therefore, the current should be uniformly distributed throughout the specimens in our experiment, with less contribution of skin effect.

6. Evaluation of the resistive-capacitive effects

The resistive-capacitive (RC) effects resulted from the stray capacitance in the circuit might lengthen the pulse width, which could influence the thermal dissipation and skin effect. However, in our experiments, these effects arise from the longer pulse should be negligible. Even if the pulse width is extended to 10 ns due to the RC effects in practice, the diffusion length in thermal dissipation is calculated to be ~1130 nm (based on the equation (4) in Supplementary Discussion 1.2), which is much larger than the maximum length of Au nanocrystals (around 100 nm); while the skin depth is calculated to be ~2.3×10^{-5} m (based on the equation (12) of Supplementary Discussion 5), which is about three orders larger than the diameters of our specimens (typically less than 20 nm, on the order of 10^{-8} m). Thus, the variation in the pulse width is insignificant on the conclusion of our experiments.

Supplementary References

1. Zhao, S. et al. Defect reconfiguration in a Ti-Al alloy via electroplasticity. *Nat. Mater.* **20**, 468-472 (2021).
2. Okazaki, K., Kagawa, M. & Conrad, H. An evaluation of the contributions of skin, pinch and heating effects to the electroplastic effect in titanium. *Mater. Sci. Eng.* **45**, 109-116 (1980).
3. Sprecher, A. F., Mannan, S. L. & Conrad, H. Overview no. 49: On the mechanisms for the electroplastic effect in metals. *Acta Metall.* **34**, 1145-1162 (1986).
4. Rupp, J. & Birringer, R. Enhanced specific-heat-capacity measurements (150--300 K) of nanometer-sized crystalline materials. *Phys. Rev. B* **36**, 7888-7890 (1987).
5. Wang, X. L., Wang, Y. B., Wang, Y. M., Wang, B. Q. & Guo, J. D. Oriented nanotwins induced by electric current pulses in Cu–Zn alloy. *Appl. Phys. Lett.* **91**, 163112 (2007).
6. Zhang, W., Sui, M. L., Zhou, Y. Z. & Li, D. X. Evolution of microstructures in materials induced by electropulsing. *Micron* **34**, 189-198 (2003).
7. Ashby, M. F. *Materials Selection in Mechanical Design (Fourth Edition)*. (Butterworth-Heinemann, Oxford, 2011).
8. Yaws, C. L. *Chemical properties handbook*. (McGraw-Hill Companies, Inc, New York, 1999).
9. Conrad, H. Electroplasticity in metals and ceramics. *Mater. Sci. Eng. A* **287**, 276-287 (2000).
10. Kittel, C. *Introduction to Solid State Physics*. (John Wiley & Sons, New York, 1976).
11. Troitskii, O. A. Effect of the electron state of a metal on its mechanical properties and the phenomenon of electroplasticity. *Strength Mater.* **9**, 35-45 (1977).
12. Hirth, J. & Lothe, J. *Theory of Dislocations*. (Wiley, New York, 1982).
13. Meyers, M. A. & Chawla, K. K. *Mechanical Behavior of Materials*, 2 edn. (Cambridge University Press, Cambridge, 2008).
14. Ogata, S., Li, J., Hirosaki, N., Shibutani, Y. & Yip, S. Ideal shear strain of metals and ceramics. *Phys. Rev. B* **70**, 104104 (2004).
15. Critchley, K. et al. Near-Bulk Conductivity of Gold Nanowires as Nanoscale Interconnects and the Role of Atomically Smooth Interface. *Adv. Mater.* **22**, 2338-2342 (2010).
16. Dimitrov, N. K., Liu, Y. & Horstemeyer, M. F. Electroplasticity: A review of mechanisms in electro-mechanical coupling of ductile metals. *Mech. Adv. Mater. Struct.* **29**, 705-716 (2022).
