# Supplementary material for: Revealing the pulse-induced electroplasticity by decoupling electron wind force
Source: Nat Commun. 2022 Oct 31;13:6503. doi: 10.1038/s41467-022-34333-2 (PMC9622885; doi:10.1038/s41467-022-34333-2)
Supplement: Supplementary file 2 — Description of Additional Supplementary Files [file 41467_2022_34333_MOESM2_ESM.pdf]

Description of Additional Supplementary Files

File Name: Supplementary Movie 1
Description: The non-directional migration behavior of incoherent twin boundary during the sequentially applied pulses of (1.0 V, 3 ns).
